# Current Perspectives on *Mycobacterium farcinogenes* and *Mycobacterium senegalense*, the Causal Agents of Bovine Farcy

**DOI:** 10.1155/2014/247906

**Published:** 2014-04-30

**Authors:** Mohamed E. Hamid

**Affiliations:** Department of Clinical Microbiology and Parasitology, College of Medicine, King Khalid University, P.O. Box 641, Abha 61321, Saudi Arabia

## Abstract

*Mycobacterium farcinogenes* and* M. senegalense* are the causal agents of bovine farcy, a chronic, progressive disease of the skin and lymphatics of zebu cattle. The disease, which is prevalent mainly in sub-Saharan Africa, was in earlier times thought to be caused by* Nocardia farcinica* and can be described as one of the neglected diseases in cattle. Some aspects of the disease have been investigated during the last five decades but the major development had been in the bacteriological, chemotaxonomic, and phylogenetic aspects. Molecular analyses confirmed that* M. farcinogenes* and* M. senegalense* fall in a subclade together with* M. houstonense* and* M. fortuitum*. This subclade is closely related to the one accommodating* M. peregrinum*,* M. porcinum*,* M. septicum*,* M. neworleansense*, and* M. alvei*. DNA probes were designed from 16S-23S rRNA internal transcribed spacer and could be used for the rapid diagnosis of bovine farcy. An ELISA assay has been evaluated for the serodiagnosis of the disease. The zoonotic potentials of* M. farcinogenes* and* M. senegalense* are unknown; few studies reported the isolation of* M. senegalense* and* M. farcinogenes* from human clinical sources but not from environmental sources or from other domestic or wild animals.

## 1. Introduction

Bovine farcy (which is caused by* Mycobacterium farcinogenes *and* M. senegalense*) is a chronic granulomatous inflammation of the skin lymphatics and draining lymph nodes of zebu cattle. It has been reported in 19 countries in Africa, Asia, Latin America, and the Caribbean with tropical and subtropical climates. Historically, it existed in a belt that extends east to include south India, Sri Lanka, and Sumatra and west to include north parts of Latin America and the West Indies but mainly dominant in the sub-Saharan African countries [1]. It was in 1888 that Edmond Nocard first isolated and described the causal agent of “bovine farcy” [2]. In his original description, Nocard [2] noted a granulomatous disease of cattle with multiple abscesses, draining sinuses, pulmonary involvement, emaciation, and eventually death. Since then, the classification of the* Nocardia* organisms has undergone several changes.

Literature on the prevalence, transmission patterns, and risk factors of bovine farcy is deficient. The disease is not included within the categories of cattle diseases in List A or List B of the OIE categorization [3] due to its characteristic that it has neither international spread nor significant mortality and morbidity at the level of a country or a zone nor an apparent zoonotic property with severe consequences. Nevertheless, cattlemen and governments in Africa believe that bovine farcy is responsible for certain economic losses as a result of damaged hides. Besides, it is public-health burden since the lymphadenitis due to farcy resembles the lesions of bovine tuberculosis in carcasses and the meat is considered inappropriate for human consumption [1].

Laboratory diagnoses are hardly ever used to make routine diagnosis and to initiate treatment. This is because of logistic and practical difficulties encountered amongst rural communities in Africa. However, laboratory diagnoses can confirm the clinical diagnosis retrospectively on tissues and purulent materials taken during treatment or during meat inspection. Apart from the reasonable use of standard smear-and-culture methods, few diagnostic tests have been developed; the molecular and serological tests have not been evaluated for reproducibility and accuracy.

## 2. Taxonomy

Bovine farcy causing actinomycetes isolated from zebu cattle in eastern Africa were found to belong to the genus* Mycobacterium *and not to* Nocardia* [4–10]. The causal agents of bovine farcy contained mycolic acids, the esters of which yielded, on pyrolysis gas chromatography, a single peak that corresponded to the C24 ester characteristic of some mycobacteria. Chamoiseau [7] suggested that these bacteria be allocated in the genus* Mycobacterium* as* M. farcinogenes*; he distinguished two subspecies,* M. farcinogenes *subspecies* tchadense* and* M. farcinogenes *subspecies* senegalense*. On the ground of their phenotypic dissimilarity, Chamoiseau [11] raised the two subspecies to species levels as* M. farcinogenes *and* M. senegalense *which have first appeared in the 1st edition of Bergey's Manual of Systematic Bacteriology [12].

It is now known that* M. farcinogenes *and* M. senegalense *can also be distinguished from one another on the basis of histopathological behavior [11], DNA relatedness [13], mycobactin contents [14], chemotaxonomic and biochemical properties [15–17], pyrolysis mass spectrometry [18], and 16S rRNA sequence data [19–23].

**Figure 1:**
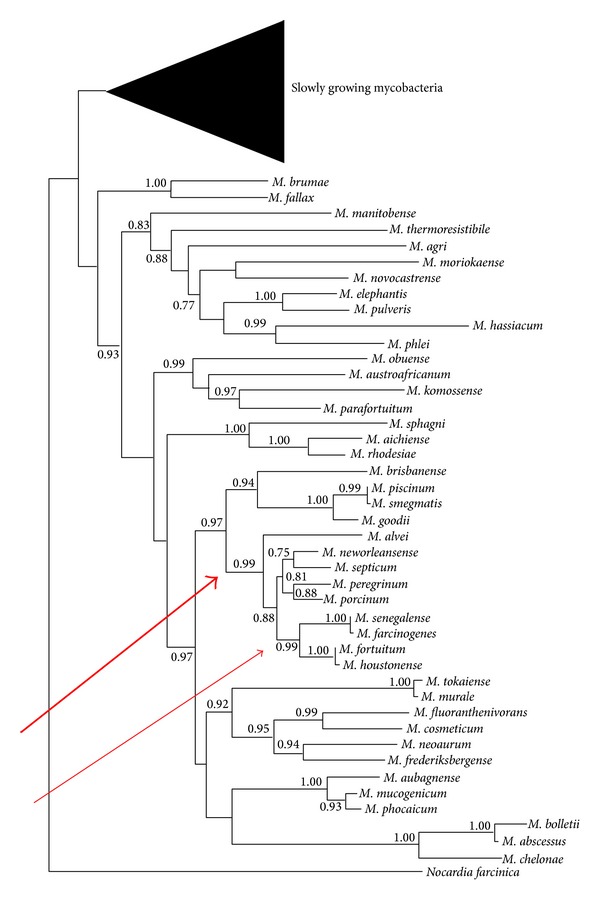
Estimate of mycobacterial phylogeny based on a multilocus seven-gene concatenate (hsp65, rpoB, 16S rRNA, smpB, sodA, tmRNA, and tuf) showing the position of* Mycobacterium farcinogenes* and* M. senegalense* within a branch that accommodates the rapidly growing nonphotochromogenic mycobacteria (thick arrow) and their close relationship to* M. houstonense* and* M. fortuitum* (thin arrow). The percentages of bootstrap values are shown next to the nodes. The tree was modified from Mignard and Flandrois [26].

Members of the two species have many properties in common both with one another and with the nonphotochromogenic rapidly growing mycobacteria, namely,* M. fortuitum *and* M. peregrinum*,* M. septicum *(sorbitol negative 3rd* M. fortuitum* biovar),* M. porcinum* [24], and, with the recently described species in the* M. fortuitum* complex,* M. boenickei*,* M. houstonense* (sorbitol negative 3rd* M. fortuitum* biovar), and* M. neworleansense* (Figure 1; [25, 26]).

*M. porcinum* is a known veterinary pathogen [27]. Phylogenetic tree of the combined rpoB + recA + soda + hsp65 + 16S rRNA gene sequences of 19 rapidly growing mycobacteria using the neighbor-joining method supported the designation of the* M. fortuitum* grouping into subclades which include* M. peregrinum *and* M. septicum; *this is joint by another subclade consisting of* M. boenickei, M. neworleansense,* and* M. porcinum *along with subclade in* M. fortuitum *and* M. houstonense, M. farcinogenes *and* M. senegalense. *Both subclades are related to another subclade of* M. farcinogenes* and* M. senegalense*. The three are related to the subclade which includes* M. fortuitum *and* M. houstonense *[28]. The 99% rpoB gene sequence similarity between* M. houstonense* and* M. fortuitum* suggested that these strains may be closely related subspecies, although* M. houstonense* showed resistance to pipemidic acid, biochemical differences such as mannitol, inositol, sorbitol, and trehalose utilization [22], and three base differences in the 16S rRNA gene sequence [25]. The isolation of* M. fortuitum* 3rd variant from cattle specimens [29] may be of epidemiologic and taxonomic implications. The* M. fortuitum* 3rd (sorbitol positive) variant is now reclassified as* M. houstonense* sp. nov. [25]. Recently, Guérin-Faublée et al. [30] described* Mycobacterium bourgelatii, *a new rapidly growing nonphotochromogenic species which they had isolated from cattle with lymphadenitis. These new isolates need to be compared with other cattle pathogens giving the similarity in both chromogenicity and the rapidly growing property.

*N. farcinica* is still though not often reported as a causal agent of bovine farcy when diagnosis based on morphological traits, which fail to discriminate* Nocardia* from other mycolic acid-containing actinomycetes, is used [31–40].

**Figure 2:**
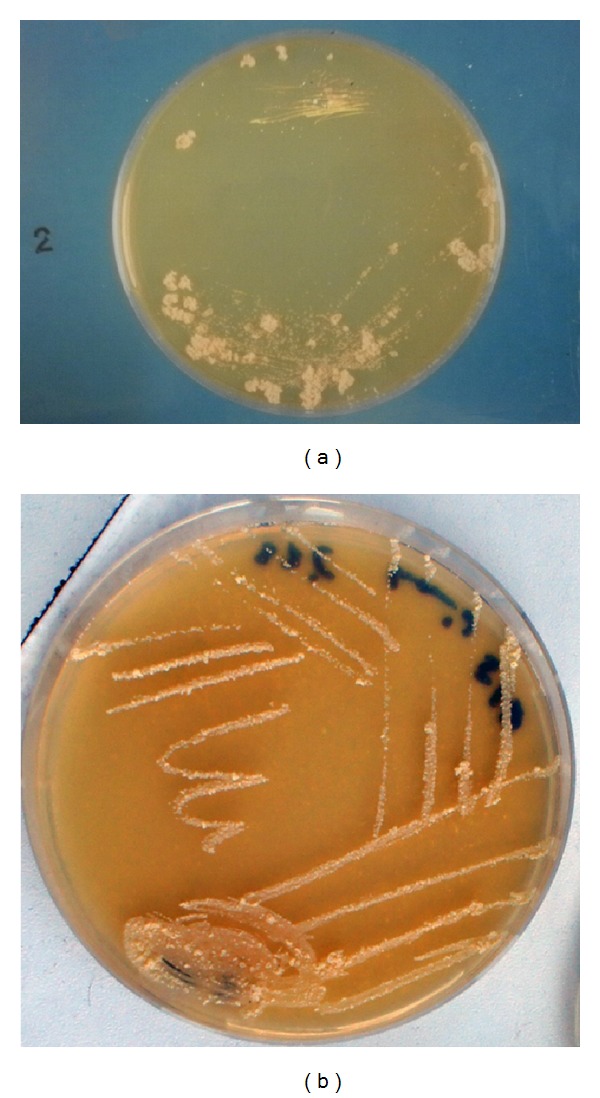
Growth of* Mycobacterium farcinogenes* on glucose yeast extract agar (a) and* M. senegalense *on glucose yeast extract malt extract agar (b) at 37°C for 7 days, showing nonchromogenic, wheat-colored rough convoluted irregular colonies.

**Figure 3:**
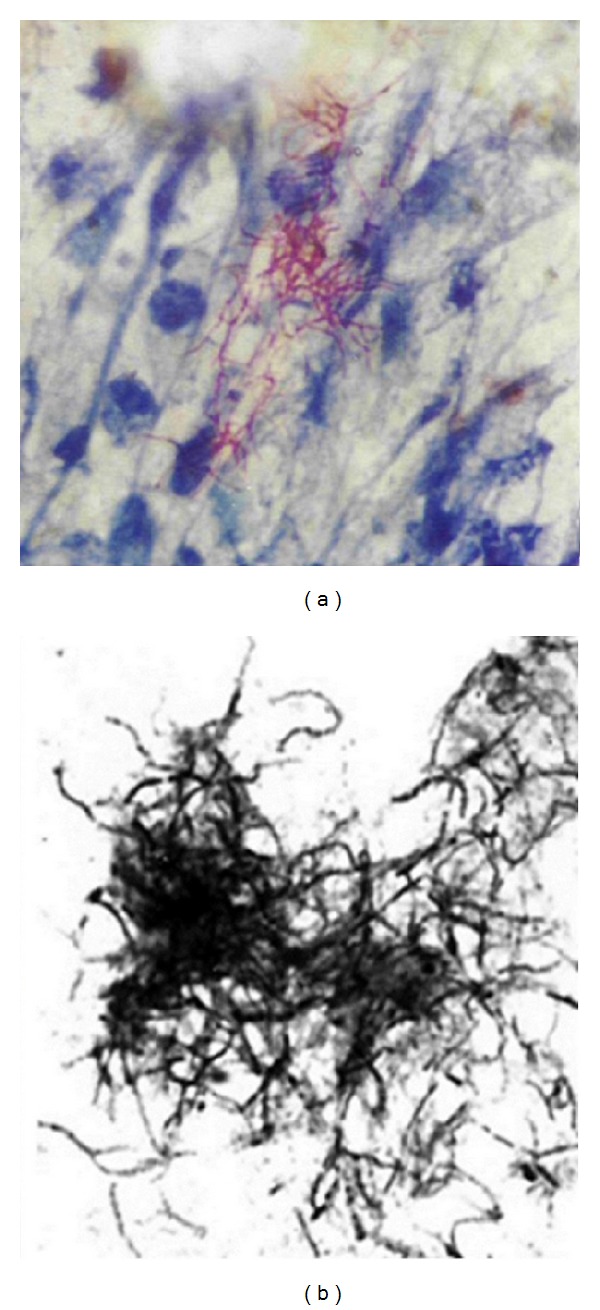
Smears made from a purulent material of* M. farcinogenes*-infected cow (a) showing acid fast branching filaments and smear made from a culture of* M. senegalense *(b). Note short or long filaments, bent and branched, in clumps or tangled lacy network which do not fragment into bacillary forms.

The conclusions from the many taxonomic studies could be summarized as follows:

1. *M. farcinogenes *and* M. senegalense *are morphologically similar to each other and to* Nocardia farcinica *(Figures 2 and 3). But* M. farcinogenes *is relatively a slow growing* Mycobacterium* compared to the rapidly growing* M. senegalense*.
2. On molecular basis,* M. farcinogenes *and* M. senegalense *are closely related to* M. houstonense. *The three species fall ina subclade including* M. fortuitum*. This subclade is closely linked to the one incorporating* M. peregrinum, M. porcinum*,* M. septicum, M. neworleansense,* and* M. alvei* (Figure 1).

## 3. Habitats

There are no reports on isolating or detecting* M. farcinogenes *or* M. senegalense *from environmental samples. Epidemiological data have not reported bovine farcy in wild and other domestic animals. The zoonotic potentials of* M. farcinogenes *and* M. senegalense *are unknown; only few reports provided evidence that* M. senegalense* [41] and* M. farcinogenes* [42] cause infections in human.

The role of ticks in the transmission of farcy is not understood. Bovine farcy lesions start at the lymph nodes (usually peripheral, femoral, or parotid) and then spread slowly via lymphatic vessel to subcutaneous tissue on the dorsal parts. In contrast, ticks feed mostly on the ventral aspects. Furthermore, tick larvae and nymphs moult before feeding on another animal; therefore, it is not feasible that the bacterium is transferred from one host to another on the outside of ticks. Although transmission of the infection by ticks under field condition has not been established, it has long been believed by locals in the Sudan that the ticks are involved. Some authors associate bovine farcy with tick infestation (notably, the ixodid tick* Amblyomma variegatum*) [40, 43–45]. Additionally, Al Janabi et al. [46] successfully transmitted* N. farcinica *(which was believed at that time to be the agent of bovine farcy) from an experimentally infected rabbit to a control one via* Amblyomma variegatum.*

## 4. Isolation and Cultivation

Lowenstein-Jensen is the medium commonly used for selective isolation of* M. farcinogenes *as for many other mycobacteria from infected materials [47]. Glucose yeast extract agar ([48]; GYEA) is used for maintenance and bench work. Modified Sauton's broth [49] is routinely used to cultivate biomass of* M. farcinogenes*,* M. senegalense, *and some other mycobacteria for chemotaxonomic studies [16, 50–52].* Mycobacterium* medium number 219 is recommended by DSMZ for routine culture [53].

*M. farcinogenes *and* M. senegalense *grow on a wide range of common synthetic media. Shigidi et al. [38] used diagnostic sensitivity test (DST) agar for culturing farcy organisms. Out of 13 diverse agar-based media,* M. farcinogenes *was found to grow particularly well on Mueller Hinton's medium followed by modified Bennett, Tryptic soya, glucose yeast extract, and DST agars [54]. A broth medium containing (g/L; w/v) yeast extract (4), glucose (15), magnesium sulphate (0.5), trisodium citrate (1.5), potassium sulphate (0.5), and ammonium ferric citrate (trace) and buffered with potassium dihydrogen phosphate (5) was formulated [55] and found to support a luxuriant growth of* M. farcinogenes *strains than media used before [49].

## 5. Morphological and Cultural Characteristics

Colonies appear after 2 to 5 days (*M. senegalense*) and 5 to 10 days (*M. farcinogenes*) at 25°C to 37°C on Lowenstein-Jensen medium. Colonies appear rough and convoluted that are firmly attached to the media. The grown colonies are usually nonchromogenic, wheat-colored. Growth on most agar-based media such as GYEA, DST agar, Tryptic Soya agar, and Mueller Hinton agar is seen as nonchromogenic irregular rough colonies (Figure 2) which are not firmly attached to the median (in contrast to their growth Lowenstein-Jensen slants) and are difficult to emulsify [11, 15, 45, 56]. These mycobacteria can be preserved for up to 10 years when cells are suspended in 20% glycerol and kept frozen at −20°C [57].

**Figure 4:**
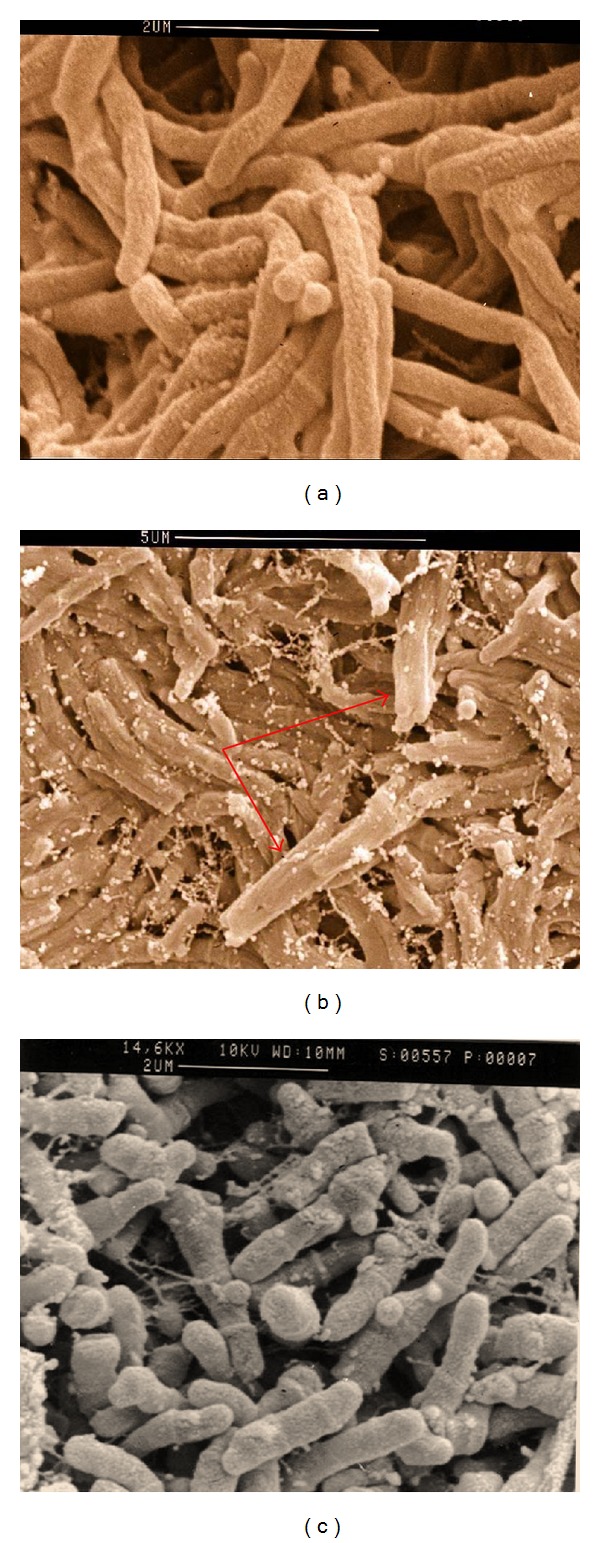
Scanning electron microscopy of* M. farcinogenes *(a),* M. senegalense *(b), and* Nocardia farcinica *(c). Note the true-nonfragmenting branched filaments in both species and the presence of “synnemata” in* M. senegalense *(arrow).

*M. farcinogenes *and* M. senegalense *have short or long filaments, bent and branched, in clumps or tangled lacy network whether seen in smears from culture or from lesions (Figure 3). These filaments do not fragment into bacillary forms and were strongly acid-alcohol fast. Scanning electron microscopy observations have confirmed the true-nonfragmenting filamentous nature of the* M. farcinogenes *and* M. senegalense* (Figure 4). It is obvious that these species could be distinguished from other mycobacteria because they form branched substrate mycelia. Moreover,* M. senegalense *exhibitsa characteristic fungal structure called “synnema” (plural synnemata) which is strand resembling stalks thread together (Figure 4).

## 6. Biochemical Features

Members of the* M. farcinogenes *and* M. senegalense *produce a positive malonamidase test, an attribute that is rarely shown by other mycobacteria. Routinely,* M. senegalense *is more active biochemically than* M. farcinogenes*. Some of the biochemical properties, enzyme profile, tolerance to chemical inhibitors, and resistance pattern to antibiotics as well as morphological and cultural characteristics of the two species are shown in Table 1.

## 7. Chemotaxonomy 

*Glycolipids and Phospholipids. M. farcinogenes *and* M. senegalense *have been found to contain trehalose dimycolate (cord factor), phosphatidylethanolamine, and phosphatidylinositol [4, 58]. Glycopeptidolipids (GPL), the so-called C-mycosides, have been found in some* M. senegalense *strains [16, 58]. Four groups of antigenic glycolipids have been detected in some* M. senegalense *strains [16]. The* M. senegalense *strains were considered to belong to two major groups. The first group which includes the majority of the strains as well as the type strain (NCTC 10956) has an alkali-stable glycopeptidolipids class of antigens [16, 59, 60]. The second group belongs to the alkali-labile acyl trehalose lipooligosaccharide class of antigens [16, 60]. The first group was found to share its properties with those described in* M. peregrinum* [16, 61] and* M. porcinum* [59], the same unusual distribution of the alaninol end of the molecules. These data reinforce the close taxonomic relationships between the three mycobacterial species and demonstrate the antigenicity of the new variants of mycobacterial glycopeptidolipids, whereas the second group has structures similar to those produced by the antigenic lipooligosaccharides of* M. fortuitum *[61, 62].

*Mycolic acids*.* M. farcinogenes* and* M. senegalense* strains contain mycolic acids that can be separated into *α*, *α*′, and epoxymycolates. Similar mycolic acid patterns are characteristic of* M. chitae, M. fortuitum, M. peregrinum, M. smegmatis*, and* M. porcinum* [63, 64]. Mycolic acids are B-hydroxy fatty acids substituted at the a-position with a moderately long aliphatic chain. The distribution of these molecules is restricted to strains in the suborder Corynebacterineae which include the genera* Hoyosella, Amycolicicoccus, Corynebacterium, Dietzia, Gordonia, Hoyosella, Millisia, Mycobacterium, Nocardia, Rhodococcus, Segniliparus, Skermania, Smaragdicoccus, Tsukamurella, *and* Williamsia* [65, 66]. These actinomycetes have an arabinogalactan-based cell wall type IV. The causal agents of bovine farcy can easily be discriminated from* N. farcinica* type strains on the basis of mycolic acid analysis.* Nocardia* species show a single mycolic acid spot on thin layer chromatography [50, 52, 67].

Members of* M. farcinogenes* and* M. senegalense*, like many other mycobacteria, undergo a characteristic cleavage reaction on pyrolysis gas chromatography. In addition to meroaldehyde, they release tetracosanoic acid (C24) as major ester fragment [52, 68]; some other mycobacteria, for example,* M. tuberculosis,* release hexacosanoic acids (C26).

El Sanousi and Tag El Din [51], using modified precipitation technique of Kanetsuna and Bartoli [69], were able to assign bovine farcy strains to the genus* Mycobacterium*. Later, Hamid et al. [16] developed a new effective mycolic acid precipitation method for the distinction between mycobacteria and other mycolic acid-containing taxa. The method was based on the precipitation of mycolic acids methyl esters in a mixture of acetonitrile and toluene (3 : 2, v/v). The method was proven to be useful, particularly to accommodate many isolates of bovine farcy to the genus* Mycobacterium* by giving copious white precipitate when acetonitrile and toluene were used to precipitate mycolic acid methyl esters.

## 8. Antigenicity and Immunogenicity

Awad and Karib [32] found that bovine farcy animals induced sensitivity to avian and mammalian tuberculins. This finding was later supported by Mostafa [70] who in addition used immunogens prepared from the causal agents of bovine farcy and it was found to give a profound reaction with high specificity and sensitivity than did the preparations from avian and mammalian strains. Magnusson and Mariat [71] have developed an immunological method based on the specificity of delayed-type skin reactions on guinea pigs for comparing* Nocardia *strains including isolates from cases of bovine farcy. The method noticeably differentiated bovine farcy strains, which formed a homogeneous group that was readily separated from reference (type) strains of* N. asteroides*,* N. brasiliensis,* and* N. farcinica*. Comparative immunodiffusion studies by Ridell and Norlin [72], Ridell [73–75], and Ridell et al. [50] indicated that the bovine farcy organisms had stronger affinity to mycobacteria than to* Nocardia*. From these studies, two serological groups were mainly identified, the first group includes the* N. farcinica* ATCC 3318, which seemed to belong to the genus* Nocardia*, whereas members of the other group including farcy strains were more closely related to* Mycobacterium* strains. The distribution of precipitinogens showed that* M. farcinogenes *and* M. senegalense *were closely related and were found to share a large number of precipitinogens. Both species shared visible precipitinogens with some other mycobacterial strains, particularly* M. fortuitum*,* M. peregrinum,* and* M. smegmatis* [75].

Using gel diffusion precipitin test and immunoelectrophoresis, Shigidi et al. [38] found that most of the strains isolated from cases of bovine farcy in Sudan reacted with antiserum from* N. farcinica* but not with antiserum from* M. tuberculosis* and* M. bovis*. In other studies, these strains were proved to be mycobacteria and were classified as* M. farcinogenes* ([16, 51]). In separate studies, when antigen prepared from whole cells of* M. farcinogenes *was tested against sera collected from animals infected with bovine farcy, only traces of agglutinin were detected [76] but limited, though sharp, precipitin lines were detected in most of the sera [52]. These two findings imply that circulating antibodies were also involved in the immunity and protection mechanism of infection with* M. farcinogenes*. Enzyme linked immunosorbent assay (ELISA) was evaluated for the serodiagnosis of bovine farcy among clinically proved cattle. Whole cell homologous suspension of* M. farcinogenes* was used as antigen and the test revealed a sensitivity of 92.7% and a specificity of 97% [77].

On the basis of antigenic cell surface glycolipids,* M. senegalense *strains were found to fall into four serological groups [16], whereas the majority of* M. farcinogenes *did not contain such antigens. The structure of the main group of* M. senegalense, *which included the type strain, was determined as glycopeptidolipids [59, 60]. These glycolipids were highly reactive to homologous sera prepared from whole cell* M. senegalense *and to lesser extent with heterologous sera from* M. peregrinum* [16, 78].

A wild strain of* M. farcinogenes* (A24) was subjected first to serial passage (20) in modified Sauton's broth then in guinea pigs. The result of the vaccination with the attenuated strain in calves revealed that 75% of calves in the vaccinated group were protected and endured the challenge infection with a virulent freshly isolated* M. farcinogenes* [79].

## 9. Molecular Analysis

DNA-DNA homology studies have indicated that* M. farcinogenes *and* M. senegalense *were separate species. According to Baess [13],* M. senegalense *is moderately related to* M. farcinogenes*,* M. fortuitum*, and to* M. peregrinum*. This fact has been further authenticated by Rogall et al. [19]. Using genus specific oligonucleotide,* M. farcinogenes *and* M. senegalense *reacted positively in the mycobacterial system [80].

Earlier sequencing of the 16S rRNA showed a close relationship between* M. chelonae*,* M. farcinogenes, M. fortuitum, *and* M. senegalense *[19, 21].* M. farcinogenes, M. senegalense, M. chelonae*,* M. fortuitum, *and* M. peregrinum* form a distinct evolutionary branch within the adaptive radiation accommodated by the genus* Mycobacterium* [19–21]. These species form the rapidly growing nonchromogenic mycobacteria.

Consequent studies indicated the close phylogenetic relationship of farcy agents to members of the* M. fortuitum *complex [81]. With the appearance of new rapidly growing species, Adékambi and Drancourt [28] accommodated these into two subclusters: (i)* M. peregrinum, M. septicum, M. neworleansense, *and* M. porcinum* and (ii)* M. farcinogenes, M. senegalense, M. houstonense, *and* M. fortuitum*. These clustering and the subclusters agree with that of Schinsky et al. [25].

16S rRNA sequences of* M. farcinogenes *and* M. senegalense *are very similar and when using the Kirschner diagnostic helix 10 and helix 18 it is not possible to differentiate between the two species (Table 2). However, when using 16S-23S rDNA spacer, Hamid et al. [23] were able to distinguish between the two species with ample number of base substitution. Two probes designed on the basis of all of the available spacer sequences were evaluated for specificity, namely, biotin–3-TCAGCCAGCATCTGTAG and biotin–3-AGGAGTCTGTGCGCTGT, as probes for the rapid diagnosis of the disease from clinical specimens or for identification of unknown strains of* M. farcinogenes *or* M. senegalense*, respectively [23].

## 10. Antimicrobial Susceptibility

Most of the* M. farcinogenes *and* M. senegalense *strains tested* in vitro *were found susceptible to cycloserine [11], dapsone [15], or amikacin, doxycycline HCl (64 *μ*g/mL), oxytetracycline HCl (64 *μ*g/mL), and paromomycin sulphate (64 *μ*g/mL) [55]. Susceptibilities to other antimicrobial agents and to various chemical agents are shown in Table 1.

## 11. Conclusions

Basic information about* M. farcinogenes *and* M. senegalense *is available in the literature. These actinomycetes are unique in their morphologies and exhibit some distinctive characteristics. These characteristics, notably, cell wall chemical markers and DNA sequence data, separate them from* Nocardia farcinica *and from closely related nonphotochromogenic rapidly growing mycobacteria. There are hardly any new reports of isolating these bacteria from cattle in recent times. On the contrary, limited numbers of reports have incriminated* M. farcinogenes *and* M. senegalense *as causal agents of human diseases.

## Figures and Tables

**Table 1:** Phenotypic characteristics of *Mycobacterium farcinogenes* and *Mycobacterium senegalense*.

Test	*M. farcinogenes *	*M. senegalense *	References*
Morphology and cultural characteristics			
Growth at 30°C	+	+	[56]
Growth at 35–37°C	+	+	[56]
Growth after 3–5 days	−	+	[11, 15, 56]
Growth after 5–10 days	+	−	[11, 15, 56]
Colony wheat-colored	+	+	[11, 15, 56]
Colony convoluted	+	+	[11, 15, 56]
Colony rough	+	+	[11, 15, 56]
Colony very rough and grainy	+	−	[11, 56]
Colony easily detached from agar	+	−	[15, 56]
Colony relatively emulsifiable	−	+	[11, 56]
Colony nonemulsifiable	+	−	[56]
Aerial hyphae sparse	+	+	[15, 56]
Aerial hyphae abundant	−	−	[15, 56]
Biochemical (enzyme) tests			
Acetamidase	+	+	[11, 15, 82]
Allantoinase	+/−	+	[11]
Arylsulphatase	−	+	[15, 76, 82]
Benzamidase	+/−	+	[11]
Catalase**	+	+	[11, 15, 56, 76, 82]
Iron citrate test	−	−	[11]
Isonicotinamidase	+/−	+	[11]
Beta-lactamase after 60 min	+	+	[15]
Neutral red test	−	−	[11]
Niacin production	−	−	[11]
Nicotinamidase	+/−	+	[11]
Nitrate reductase	+	+	[11, 15, 76]
Nitrophenol oxidase	−	−	[15]
Salicylamidase	+/−	+	[11]
Urease	−/+	+	[11, 15, 82]
Urease	−	ND	[76]
Fluorogenic enzyme tests (cleavage of 4-methylumbelliferone glycosides substrates (4MU))			
4MU-*α*-L-arabinofuranoside	+	+/−	[56]
4MU-*α*-L-arabinopyranoside	+	+/−	[56]
4MU-*β*-D-fucoside	+	+/−	[56]
4MU-*β*-D-galactoside	+	−	[56]
4MU-*β*-D-glucoside	−	+	[56]
4MU-*β*-D-glucoside	+	+	[56]
4MU-p-guanidinobenzoate (HCl)	+	+	[56]
4MU-*α*-D-mannopyranoside	−	+	[56]
4MU-*β*-D-ribofuranoside	+	+	[56]
Degradation tests			
Aesculin	+	+	[56]
Arbutin	+	+	[56]
Casein	−	−	[56]
Elastin	−	−	[56]
Guanine	−	−	[56]
Hypoxanthine	−	−	[56]
Keratin	−	−	[56]
Testosterone	+	+	[15]
Tyrosine	−	−	[15, 56]
Tweens	+	+	[56]
Xanthine	−	−	[56]
Growth in the presence of (%)			
Cobalt chloride (0.005)	+	+	[56]
Copper sulphate (0.01)	+	+	[56]
Crystal violet (0.001)	−	−	[15]
Ferrous sulphate (0.01)	+	+	[56]
Hydroxylamine HCl (0.05)	−	−	[56]
Lead acetate (0.01)	+	+	[56]
o-Nitrobenzoic acid (0.05)	−	+	[56]
Oleic acid (0.25, v/v)	+	+	[56]
Phenol (0.01)	+	+	[56]
Phenol (0.1)	−	−	[15, 56, 82]
Phenyl ethanol (0.02%)	−	+	[15, 82]
Potassium tellurite (0.04%)	−	+	[15, 82]
Potassium tellurite (0.5)	−	−	[56]
Pyronin G (0.1)	−	−	[56]
Sodium azide (0.005)	−	+	[56]
Sodium azide (0.01)	−	−	[15, 82]
Sodium chloride (5)	−	−	[56]
Sodium chloride (5%)	−	+	[15, 82]
Sodium deoxycholate (0.01)	−	+	[56]
Sodium nitrate (1)	−	−	[56]
Sodium salicylate (0.1)	−	+	[56]
Sodium selenite (0.001)	−	−	[56]
Thallous acetate (0.05)	−	−	[56]
Tetrazolium chloride (0.01)	−	−	[56]
Toluidine blue (0.03)	−	+	[56]
Teepol HB6 (0.05, v/v)	−	−	[56]
Zinc chloride (0.005)	−	−	[56]
Zinc chloride (0.01)	−	−	[56]
Growth at			
45°C	−	−	[15, 76]
pH4	−	−	[15]
pH5	−	+	[15, 82]
pH10	−	−	[15]
Survival at 60°C for 4 hours	−	−	[15]
Resistance to antibiotics and antibacterial agents (*μ*gmL-1)			
Amikacin (2)	−	−	[55]
p-Aminosalicylic acid, Na salt (64)	−/+	+	[11, 55]
Amoxicillin (64)	+	+	[55]
Ampicillin (64)	+/−	+	[55]
Capreomycin sulphate (10)	−	+	[15]
Capreomycin sulphate (10)	−/+	−	[55]
Cephaloridine (64)	+	+	[55]
Cephapirin Na salt (64)	+	+	[55]
Chlortetracycline HCl (64)	−/+	−/+	[55]
D-Cycloserine (2)	+	+	[55]
Cycloserine	−	−	[11]
Dapsone (16)	+	+	[55]
Dapsone (100)	−	+	[15, 82]
Doxycycline HCl (8)	−	+/−	[55]
Doxycycline HCl (64)	−	−	[55]
Erythromycin (64)	−	+	[55]
Ethambutol HCl (4)	+	+	[15, 55]
Ethambutol HCl (64)	−/+	+	[15, 55, 82]
Ethionamide (5)	+	+	[11, 55]
Gentamycin sulphate (128)	−	−	[55]
Isoniazid (2)	+	+	[15, 55]
Kanamycin sulphate (2)	+	+	[55]
Kanamycin sulphate (16)	−	−	[11]
Lividomycin sulphate (16)	−	−	[55]
Lysozyme (50)	+	+	[15]
Lincomycin HCl (64)	+	+	[55]
Nalidixic acid Na salt (64)	−	+	[55]
Novobiocin (64)	+/−	+	[55]
Neomycin sulphate (128)	−	−	[55]
Oleandomycin phosphate (64)	−	+	[55]
Oxytetracycline HCl (64)	−	−	[55]
Paromomycin sulphate (64)	−	−	[55]
Penicillin (66 IU/mL)	−	+/−	[15]
Polymyxin B sulphate (64)	−	+	[55]
Prothionamide (10)	+	+	[15]
Rifampicin (16)	+	+	[55]
Rifampicin (20)	+	−	[15]
Streptomycin sulphate (1.6)	+	+	[11, 55]
Streptomycin sulphate (64)	−	+	[15]
Sulphamethazine (1.6)	+	+	[55]
Thiacetazone (10)	+	−/+	[55]
Trimethoprim + sulphamethoxazole (8)	+	+	[55]
Vancomycin HCl (64)	−	−	[55]
Viomycin sulphate (64)	−	−	[11, 55]
Growth on sole carbon source (1%)			
Acetamide	+	+	[56]
Acetic acid (Na salt)	+	+	[56]
Butane 1,3 diol	+	−	[15, 82]
Butane 2,3 diol	−	+	[15, 56, 82]
Ethanol	−	−	[15, 82]
Fructose	−	+	[56]
D(+)Galactose	−	−	[56]
D-Gluconic acid	+	+	[56]
D(+)Glucosamine HCl	−	+	[56]
Hippuric acid (Na salt)	−	−	[56]
Lactic acid (Na salt)	−	+	[56]
Malonic acid (Na salt)	−	−	[56]
Maltose	−	−	[56]
Mannitol	−	+	[56]
Oxalic acid (Na salt)	−	−	[56]
1,2-Propanediol	−	+	[56]
Pyruvic acid (Na salt)	−/+	+	[56]
D(+)Raffinose	−	−	[56]
Rhamnose	−	+	[56]
Rhamnose	−	−	[15, 82]
Salicin	−	+	[56]
Sucrose	+	−	[56]
Tartaric acid (Na salt)	−	−	[56]
Trehalose	−	+	[56]
D(+)Turanose	−	−	[56]

*References: Chamoiseau [11]; El  Sanousi et al. [76]; Ridell and Goodfellow [15]; Ridell et al. [82]; Hamid [56]; Hamid and Goodfellow, [55]; **slide catalase test.

**Table 2:** Comparison of 16S rDNA signature sequences. The alignment comprises the two variable regions found in the 16S rRNA genes of selected members of species closely related to *M. farcinogenes *and *M. senegalense*; “-” indicates identity.

	16S rRNA position number 177 (*E. coli* position)	16S rRNA position number 254 (*E. coli* position)
*M. fortuitum *	C GAAT ATGACCAC GCGCTTCAT GGTGT	TTGGTGGGG TAATGGCCT AC
*M. houstonense *	–G– -G––– –––— –—	–––— –––— –
*M. senegalense *	–G– -G––– –––— –—	–––— –––— –
*M. farcinogenes *	–G– -G––– –––— –—	–––— –––— –
*M. boenickei *	–— -G––G- –T––– –G–	–––— –––— –
*M. neworleansense *	–— -G––G- –T––– –G–	–––— –––— –
*M. porcinum *	–— -G––G- –T––– –G–	–––— –––— –
*M. septicum *	–— –––G- –A––C- –—	–––— –––— –
*M. peregrinum *	–— –––G- –A––C- –—	–––— –––— –
*M. alvei *	–— ––—T –A––C- –—	–––— –––— –
